# Complete Genome Sequence of the Lumpy Skin Disease Virus Isolated from the First Reported Case in Greece in 2015

**DOI:** 10.1128/genomeA.00550-17

**Published:** 2017-07-20

**Authors:** Eirini I. Agianniotaki, Elisabeth Mathijs, Frank Vandenbussche, Konstantia E. Tasioudi, Andy Haegeman, Peristera Iliadou, Serafeim C. Chaintoutis, Chrysostomos I. Dovas, Steven Van Borm, Eleni D. Chondrokouki, Kris De Clercq

**Affiliations:** a National Reference Laboratory for CaPVs, Department of Molecular Diagnostics, FMD, Virological, Rickettsial & Exotic Diseases, Athens Veterinary Center, Ministry of Rural Development and Food, Athens, Greece; b Molecular Platform, Veterinary and Agrochemical Research Centre, Ukkel, Belgium; c Unit Vesicular and Exotic Diseases, Veterinary and Agrochemical Research Centre, Ukkel, Belgium; d Diagnostic Laboratory, School of Veterinary Medicine, Faculty of Health Sciences, Aristotle University of Thessaloniki, Salonika, Greece

## Abstract

Lumpy skin disease virus (LSDV) causes an economically important disease in cattle. Here, we report the complete genome sequence of the first LSDV isolate identified in mainland Europe. LSDV isolate Evros/GR/15 was isolated from the first cases reported on 18 August 2015 in the Evros region, Greece.

## GENOME ANNOUNCEMENT

Lumpy skin disease (LSD) is an economically important disease in cattle that is caused by lumpy skin disease virus (LSDV), a member of the *Capripoxvirus* (CaPV) genus. Historically restricted to Africa, the disease spread to the Middle East in 2012 and Turkey in 2013 (1). In August 2015, the first LSD cases in mainland Europe were observed in Greece in the Evros region at the European border with Turkey (2). The disease has been spreading into Europe ever since (3, 4). Here, we report the complete genome sequence of the LSDV isolated from the first outbreak in mainland Europe (isolate Evros/GR/15).

The LSDV isolate Evros/GR/15 was isolated from a biopsy tissue specimen as previously described (5). DNA was purified from cell culture supernatant using a Puregene Core Kit A (Qiagen) according to the manufacturer’s instructions. Presequencing enrichment was performed through an in-house long-range PCR method covering the entire genome with 23 overlapping amplicons of ~7.5 kb. In order to distinguish the short sequences from both inverted terminal repeats (ITR), two libraries, each compromising an equimolar pool of 12 PCR amplicons corresponding to half of the CaPV genome, were prepared using a Nextera XT DNA library preparation kit (Illumina) with 1 ng of input DNA, according to the manufacturer’s instructions. Sequencing was performed at the Genomics Core UZ Leuven (Leuven, Belgium) using a MiSeq reagent kit version 3 (Illumina) with 2- × 300-bp paired-end sequencing on a MiSeq Benchtop Sequencer (Illumina). The quality of the raw data was assessed using FastQC v0.11.3 (http://www.bioinformatics.babraham.ac.uk/) and the reads were trimmed using Trim Galore! v0.3.8 (http://www.bioinformatics.babraham.ac.uk/) based on quality (Q score > 30) and length (length >80 bp, 5′ clip for R1 and R2 = 20). The trimmed reads were *de novo* assembled into a single contig using ABySS v1.9.0 with optimized k values and a subsample of 20,000 paired-end reads (6). The contigs from both libraries were manually merged into a single sequence. Discrepancies with previously published LSDV genomes were confirmed by Sanger sequencing. The protein-coding genes were predicted by GATU relative to the LSDV field isolate Neethling Warmbaths LW sequence (AF409137) (7, 8).

The reads obtained from the amplicon sequences of LSDV isolate Evros/GR/15 were assembled into a double-stranded, linear DNA contiguous sequence of 150,554 bp, with an average G+C content of 25.89%, evenly distributed. Evros/GR/15 contains a 145,935-bp central coding region flanked by two ITRs of at least 2,190 bp. The Evros/GR/15 isolate shares 99.8% homology with the LSDV field isolate Neethling Warmbaths LW. A total of 24 single nucleotide polymorphisms (SNPs) and 14 small indels were identified when compared to the Neethling Warmbaths genome. Nine SNPs were nonsynonymous, resulting in amino acid changes in the proteins encoded by genes LD005, LD006, LD017, LD042, LD054, LD094, LD126, and LD128. Four of the small indels were coding and causing either the elimination of a single amino acid (LD096) or frame shifts in the encoded proteins (LD013a, LD026a, and LD144).

### Accession number(s).

The complete genome sequence of the LSDV isolate Evros/GR/15 has been deposited in GenBank under accession number KY829023.
